# Provider perspectives on emotional health care for adults with type 2 diabetes mellitus in the Dominican Republic

**DOI:** 10.1371/journal.pgph.0000537

**Published:** 2022-11-09

**Authors:** Deshira D. Wallace, Nastacia M. Pereira, Humberto Gonzalez Rodriguez, Clare Barrington

**Affiliations:** 1 Department of Health Behavior, University of North Carolina at Chapel Hill, Chapel Hill, North Carolina, United States of America; 2 School of Medicine, University of North Carolina at Chapel Hill, Chapel Hill, North Carolina, United States of America; Bangladesh University of Health Sciences, BANGLADESH

## Abstract

The emotional burden of type 2 diabetes mellitus (T2D) can complicate self-management. Exploring the feasibility of mental and physical health co-management in limited-resourced settings is needed. Thus, we assessed providers’ awareness of the emotional burden their patients experience and their roles in supporting their patients with T2D. We conducted a formative qualitative study using in-depth interviews with 14 providers, including physicians, nurses, and community health workers recruited at two rural health clinics in the Dominican Republic. We coded transcripts using inductive and deductive codes and developed themes through iterative comparative analysis. All providers recognized that patients experience an emotional burden managing life with T2D. Some providers viewed the provision of emotional support as integral to their role and believed that they could do so. Others viewed it as the responsibility of the family or expressed the need for additional guidance on how to provide emotional support. Providers also identified several barriers to integrating emotional support into routine clinical care including personality characteristics, lack of training, and insufficient staffing. While providers recognize the need for emotional support, they identified individual, clinical, and systems-level barriers. Strategies to address these barriers include training specific providers on emotional support provision, balancing workload, and building or strengthening referral systems.

## Introduction

As the prevalence of type 2 diabetes mellitus (T2D) increases globally, there is a need to develop low-cost interventions to improve and sustain diabetes self-management and overall wellbeing [1]. Managing T2D requires modifying and adhering to lifelong behaviors including medication use, healthy eating, and physical activity. However, difficulty coping with and adapting to a diabetes diagnosis and barriers to accessing care, among other factors, can complicate adherence to self-management and lead to diabetes-related distress, which encompasses stress, anxiety, sadness, and depressive symptoms [2, 3]. Both diabetes and non-diabetes related distress have been associated with poor adherence to T2D self-management behaviors, which leads to poorly controlled blood glucose levels and complications, such as kidney failure or nerve damage [4–6]. Therefore, it is important to address stress and overall emotional health as part of T2D management.

Compelling evidence supports the benefits of low-cost, community-based interventions, that provide social support for healthy eating, physical activity, and other health behaviors to improve T2D and associated clinical outcomes [7–9]. Such interventions generally involve family, peers, and community health workers as sources of emotional, material, instrumental, and informational support [10–12]. For example, Latinos with T2D living in Chicago, Illinois were enrolled in a program that focused on addressing T2D-related distress called Compañeros en Salud, which involved regular phone calls, and group classes and activities as a means of providing instrumental and emotional support [7]. Participants in the program had a significant decline in HbA1c levels over their two years of participation [7]. Although there is evidence of the importance of providing emotional support to improve diabetes-related outcomes, healthcare providers including nurses and physicians, largely focus on providing informational support in the form of health education about diabetes self-management and less on emotional support and diabetes-related stress [13–15].

We sought to improve understanding of the perspectives of physicians, nurses, and community health workers (hereafter known as providers to maintain confidentiality) on the emotional burden of diabetes management, and their capacity to support patients in identifying managing and reducing associated stress, anxiety, or depressive symptoms. We explored three primary questions: 1) How do providers describe and understand the emotional burden associated with living with T2D? 2) How do providers describe their role in supporting the emotional health needs of patients? and 3) What barriers exist to integrating emotional support into routine clinical care?

## Materials and methods

### Study setting

We conducted this formative qualitative study in two rural clinics in the central valley region of the Dominican Republic between May and July 2018. An estimated 8.1% to 9.3% of Dominicans live with T2D, and prevalence of T2D is estimated to rise to 10.3% by 2045 [16]. Qualitative studies conducted in the Dominican Republic found that diabetes-related stress begins at diagnosis and persists throughout the self-management process [13]. Furthermore, people with T2D reported that they often experienced a significant emotional burden as a result of their T2D; however, they lacked sufficient emotional support to cope with this emotional burden [13, 17, 18].

Access to primary and specialized diabetes care is limited in the rural areas of the Dominican Republic. To address this gap, in 2010 a US-based non-profit group, Chronic Care International (CCI) and a Dominican-based non-governmental organization, Institute for Latin American Concern (ILAC) partnered to develop a community-based diabetes and hypertension program in two rural clinics with the intent of improving health care quality and outcomes [19]. Across the two clinics there are three Dominican physicians, two nurses, nine community health workers (locally referred to as *cooperadores*), and one program manager. Patients receive free consultations with a physician, medications, and education on T2D self-management from the *cooperadores*. In addition, nurses and *cooperadores* collect patient vitals (e.g., blood pressure, weight, and fasting blood glucose) and visit patient homes to support self-management using problem solving and goal-setting techniques. At the time of this study, the clinics served over 1,000 patients living with type 2 diabetes mellitus.

### Study team

Our study team consisted of graduate students in public health, a research coordinator, and the principal investigator, who are all fluent in Spanish. The first, second, and third authors are of Latin American descent. The senior author has worked with the community for over 20 years and the study team has engaged in collaborative research with the clinic teams for 5 years. This familiarity with the context and time spent in the community facilitated our access to participants and our ability to reflect on participant narratives throughout the data collection and analysis processes. The primary interviewer resided in one of the clinic communities during data collection, which provided the opportunity for observing the overall context of people’s lives and aided in interpretation of our findings.

### Participants & data collection

We purposefully sampled providers from both participating clinics as they were information-rich informants based on their engagement with individuals with T2D. All providers (n = 14) from both clinics agreed to participate including three doctors, two nurses, and nine *cooperadores*. Participants’ mean age was 52 and they had worked at the clinic for a range of 1–8 years. Informed by our past research in this setting [13, 17, 18], we wrote the interview guide in Spanish and organized it according to the study’s three main topics: 1) how providers described the emotional burden of diabetes; 2) how they described their role in supporting the emotional health needs of patients; and 3) barriers to integrating emotional support into routine clinical care.

After obtaining verbal informed consent, we conducted audio-recorded, semi-structured in-depth interviews in-person, in Spanish, across various locations including the clinics, providers’ homes, and a community preschool. We completed fourteen initial in-depth interviews and six follow-up interviews for a total of 20 interviews. Follow-up interviews were conducted to further explore salient themes that arose during initial interviews. The fourteen initial interviews ranged from 25–93 minutes (average 53 minutes). The six follow-up interviews ranged in length from 8–28 minutes (average 18 minutes). Interviews were transcribed verbatim by a university-educated Dominican psychologist. Participants received an incentive worth USD $5. This study, including informed consent materials, was approved by the Institutional Review Board at the University of North Carolina at Chapel Hill.

### Data analysis

Data analysis involved a multi-step iterative process informed by Thomas’ [20] inductive approach. Following each interview, we completed field notes describing the interviewer-participant dynamic, the setting in which the interview took place, and salient themes that arose during the interview. Throughout data collection, field notes were used to guide revisions to the interview guide and to probe on recurring themes in subsequent interviews.

Preliminary analysis involved reading Spanish-language transcripts and checking transcription accuracy against the audio files and triangulating against field notes and observations [21]. These activities informed the development of a codebook, which was used to examine the three primary study questions and additional inductive themes such as “causes of stress” and “familial conflict.” Using Dedoose 8.1 (Los Angeles, CA), an online qualitative analysis software program, we coded each transcript, observed code co-occurrences, and created matrices to help organize illustrative quotes and explore connections across themes. After the initial round of coding and matrix creation, we revised the codebook and recoded each transcript to capture previously unidentified themes. We then examined patterns across participants to assess their perspectives of the emotional burden of living with T2D. All data were analyzed in Spanish; however, all quotes presented below were translated by our bilingual study team.

### Ethics statement

This study was conducted according to the guidelines of the Declaration of Helsinki. We did not include participant information alongside quotes to protect confidentiality. As per approved procedure, verbal consent was obtained from participants prior to data collection. The providers in the study were made aware of the study goals and the privacy of all participants has been protected.

## Results

We first present providers’ perspectives on the emotional burden of living with T2D followed by their perceptions of their roles and responsibilities in alleviating this burden, and finally the perceived challenges to integrating emotional support into the existing clinic structure. To protect participant confidentiality, we do not present identifying information about participants (e.g., gender, occupation, age) and refer to all participants as “providers”. Overall, providers recognized the emotional burden associated with patients’ T2D self-management. However, they offered varying levels of emotional support to patients and provided contrasting viewpoints on their role and capacity in providing emotional support. Some providers viewed the provision of emotional support as integral to their role and believed themselves to be fully equipped to do so. Others, however, viewed providing emotional support as the responsibility of the family or expressed the need for additional guidance on delivering stress management techniques as a means of providing emotional support. Finally, providers identified several factors as barriers to integrating emotional support into routine clinical care.

### Perceptions of the emotional burden of diabetes

Providers were keenly attuned to the emotional burden of T2D among their patients. The sources of stress providers listed were in close alignment with how patients described their stressors in previous research [13, 17]. Primarily, providers identified sources of stress related to diagnosis, long-term management, and interpersonal issues within the family. Related to diabetes diagnosis, providers described that the emotional burden their patients felt came from the uncertainty about whether they would be able to live a “normal life” and fear of falling ill or premature death.

There are many patients that when you tell them they have diabetes for the first time they think it’s a disease that is going to end [or kill] them. For example, they say, "I will not be able to eat anything, I cannot carry on a normal life […] oh, I cannot eat anything, and it’s true that I’m going to die.” They believe that because they already have diabetes that they have already lost. (Provider 1)

As described by providers, the notion of T2D disturbing patients’ “normal” lives was a common source of stress. To assuage the stress of the unknown, providers primarily worked to educate their patients and dispel inadequate or misinformation.

In addition to fear of morbidity and mortality, providers explained that their patients experienced stress related to adherence to dietary recommendations:

There is another thing that can cause stress. You know that there is a conflict between a patient with diabetes and eating, why? Because one of the causes that leads to diabetes is disordered eating. Disordered eating is related to what your palate likes the most, what you like the most. Then when you come to the doctor, the doctor asks you to control what you eat, yet most of the things that doctors recommend are the very things you do not like. When what’s prohibited are the majority of the things that you like, then that is a burden that you assume. ‘What am I going to eat now; if I do not like what they are telling me to eat?’ This [conflict] stresses the patient.” (Provider 2)

Patients’ stress related to engaging in recommended dietary behaviors was viewed as both the consequence of limited access to healthy foods as well as a conscious decision by patients to continue eating their preferred, yet non-recommended, foods. While both aspects of patients’ dietary challenges were acknowledged, the latter reflects that providers believed patients were at least partially responsible for their T2D and subsequent struggles to manage their condition because of their food preferences. Providers described how the patients’ emotional burden changed depending on their ability to effectively manage their T2D. For example, patient emotional burden reduced once patients established and were able to maintain a routine for their self-management. However, there were examples of patients’ emotional burden heightening as they tried to change their diets to recommended foods, yet they encountered structural barriers such as poor access due to lack of availability or high cost.

The clinic environment also contributed to the emotional burden experienced by patients. Specifically, providers perceived that their patients experienced stress when they came in for appointments and were told by providers that they did not achieve desired improvements in their clinical outcomes despite their best efforts to make appropriate lifestyle changes. Providers acknowledged that patients felt frustrated making substantial lifestyle changes that were not always reflected in their glucose levels. Providers shared that the uncertainty around being able to successfully manage their glucose levels lead some patients to believe that having diabetes “is worse than having HIV.”

Finally, family-level factors, specifically disruptions in interpersonal relationships and lack of familial support with T2D self-management, were identified as salient sources of stress across every interview. While providers believed that many patients received support from their families, they also believed that patients struggled because their families were not concerned enough or did not do enough to support patients’ diabetes management. One provider offered up common grievances they heard from patients regarding the support received from family:

Patients may say: ‘my family does not understand me’, ‘I do not feel well because my family does not understand me’ […] ‘I live with this disease and nobody understands me’ or ‘my family does not care if I have diabetes’, or ‘they do not take into account that I am a sick person.’ If the patients are women, then they usually mention how they have to do everything [in the home], and that nobody helps them. Specifically, how their husbands do not think that they have to eat a specific diet, but they actually need to. (Provider 3)

In contrast, reflecting the high levels of migration from these communities, providers also noted that some patients experienced stress due to being alone and isolated from family.

Other times [it is] because they have no family and live alone, other times, for example, a patient told me that her daughter left the country and left her two children with her. [The patient] was alone, sick, with two children, with two grandchildren and she was emotionally distressed.” (Provider 4)

Notably, the quotes above highlight an important gendered pattern of women feeling less supported by their male partners in their diabetes management. Gender also played a role in inequitable caretaking responsibilities and social isolation, with participants noting that their women patients were often burdened by having to care for their families while not receiving the same levels of family support as compared to the men in the program. Participants shared that some patients may forego self-management regimens or struggle to engage with treatment regimens because they must take care of loved ones with little, or sometimes no support from other family members.

### Perceived roles

Providers expressed diverging viewpoints on their role and capacity to address the emotional burden related to T2D experienced by their patients. Most commonly, providers described their primary role as providers of instrumental and informational support for their patients, which is congruent with previous findings from patient interviews [13]. Instrumental support arose in descriptions of their job responsibilities with examples including dispensing patients’ medications, raising funds to support clinic functions, or accompanying patients to medical visits outside of the clinic. Informational support consisted of educating patients on the three fundamental aspects of diabetes self-management (i.e., medication adherence, healthy eating, and physical activity). Providers regarded education as a critical component of patient support, with one provider asserting that education was the “most important” aspect of patients’ care needs and would “solve all the problems [they face].”

In addition to instrumental and informational support, providers also recognized the importance of emotional support as an essential component of the clinic services and their roles, with one provider saying, “We are the patients’ mantel of tears […] As I always say, the doctor is more of a counselor than a doctor.” (Provider 5) They further asserted, “if [we have] 100 patients, all 100 will need some form of counseling” in the form of encouragement and emotional support. Importantly, they believed that “A patient cannot improve if you do not look at what’s going on with them emotionally,” emphasizing the importance of addressing patients’ contexts and acknowledging the multiple factors affecting their ability to manage their condition. In fact, three providers spoke of visiting patients at their homes to offer emotional support. One provider in particular spoke about the home visitation program, which was developed for patients who were struggling with controlling their T2D, and noted that it was in these home visits that they could support patients when “they experienced a state of depression or felt emotionally burdened” as well as support patients and their families diabetes management strategies (Provider 4).They explained that this individualized follow-up was logistically challenging; however to account for this, they engaged family and neighbors as other sources of support for the patient.

Some providers appreciated the importance of emotional support and felt comfortable providing it; however, many expressed uncertainty or discomfort and believed that emotional support should primarily come from family, “trusting in God”, or mental health professionals. One provider specified why the family’s role, while variable, was critical in providing emotional support:

When patients feel depressed, when their families do not help them, don’t counsel them, don’t support them, we do support patients. But it’s not the same when they are here with us compared to when they are in their homes, because they are only here for a couple hours, but they’re in their homes daily. So, they need their family members to help them, to support them, and to counsel them. (Provider 6)

Although family were deemed as essential sources of emotional support, providers acknowledged their roles in providing support to their patients as well. Additionally, participants noted other clinic staff who they perceived possessed the appropriate disposition to provide emotional support. Specifically, they spoke of the qualities these team members. As one provider mentioned “a person who is humble…who you can talk to and listens to you, makes time for you and does not make you feel rushed,” as important characteristics that best equipped providers to support patients’ emotional needs.

### Barriers to supporting patients

While none of the participants expressed total unwillingness to provide emotional support, several described important challenges that hindered their ability and preparedness to do so. Across both clinics, the broad consensus was that inadequate training, lack of trained personnel, and limited contact with patients beyond the clinical encounter challenged their ability to adequately support the emotional needs of their patients.

[We need] a workshop or something like that where we can learn more about how to help people who have [emotional and mental health] concerns. (Provider 7)

Providers noted that they were trained to provide educational talks around T2D self-management and specific clinical services for their patients, and that they were not trained in providing emotional and mental health support. For example, educational talks primarily covered topics central to T2D self-management, such as glucose control, healthy eating, and exercise. Clinic services were often directly related to measurable outcomes in diabetes care, specifically measuring high blood pressure, lipids, and blood glucose levels, or distributing medications and tracking educational materials discussed during appointments. For some, activities outside of the scope of clinic tasks caused feelings of discomfort among providers, as expressed by one:

Because I do not know how to express it well, how to give that spirit that they need […] my job is to talk to them about A1C [blood glucose], I tell them: “you have to feed yourself, do your exercises.” (Provider 8)

This quote highlights how this provider was more comfortable providing informational rather than emotional support. In addition to inadequate training on the provision of emotional support, providers also cited limited time during clinical encounters coupled with limited exposure to patients outside the clinic as another barrier. Lastly, providers expressed concern over the limited staff available to provide adequate levels of care should they take on a more active emotional support role. This was a particularly relevant concern as providers noted an increasing patient population requiring support for their T2D management. However, several providers mentioned times when they provided emotional support to patients and identified certain providers who excelled in doing so, or the necessary characteristics for those who can most successfully provide emotional support, specifically patience, understanding or empathy, and a willingness to be patient-facing.

## Discussion

This study provides insights into providers’ perspectives on the emotional wellbeing of patients with T2D in the rural Dominican Republic as well as their role in addressing emotional health needs. We found that providers’ perceptions of the emotional burden of T2D was consistent with patient’s narratives of stress described in past research, indicating that they appreciated that stress was a salient issue among patients [17]. Within the context of the T2D clinics in the DR, patients have previously reported experiencing increased distress related to managing their T2D and a desire for more emotional support from providers [13]. This agreement between provider and patients in terms of their appreciation of the emotional burden of T2D could be an enabling factor for future interventions that aim to support emotional wellbeing.

The American Diabetes Association recommends assessment and referral, as needed, for mental health concerns, such as diabetes-related distress, anxiety, and depression [22]. However, this recommendation requires existing referral systems or mental health service integration in primary care settings, which is not widely available or feasible, especially in low and middle income countries [23]. In clinic environments that are not tied to referral systems, delivery of comprehensive diabetes self-management support systems, such as mental health resources, may be uncoordinated meaning that health care is provided by separate systems with little communication and follow-up between providers.

A recent review by Werfalli et al. [24] found that in low- and middle-income countries task shifting may be a potential solution to limited referral systems. In task shifting, a task normally performed by physicians is transferred to a health professional with different education and training or to a person specifically trained to perform a specific task, without having formal health education [24]. Task shifting can involve community health workers or patient peers working with patients to manage chronic conditions and overall wellbeing. For example, a Guatemalan program identified and trained community health workers to provide education and support to Mayan adults with T2D [25]. In addition to diabetes education, each community health worker had a caseload of 15–20 patients and held diabetes clubs to create a social network of support and also met with patients individually to provide emotional support to deal with the stigma and sadness they experienced with living with T2D [25]. At follow-up, HbA1c values significantly decreased, and qualitative findings from focus groups with participants found improved coping mechanisms, empowerment, and experiences with social support [25]. While programs like this one in Guatemala have significantly reduced distress and improved wellbeing [26], some providers in this study were apprehensive in being tasked to provide this form of support due to a lack of training. Programs that have effectively addressed emotional and mental health needs of patients explicitly trained community health workers on the provision of mental, emotional, and social health [27]. Rothschild et al. [27] assessed a community health worker-based intervention among Mexican Americans with T2D who taught general self-management skills, problem solving, seeking social support from family and friends, and managing stress.

Although task shifting could improve integration of emotional support into clinical care, there is a need to develop and strengthen capacity. Providers in this study identified their lack of training in mental health as the main barrier to providing such support to patients, which has been documented in studies with providers in Mexico [28] and the DR [29]. Of note, at the time of this study, the Chronic Care International multi-disciplinary team was continuing a capacity building process with these Dominican providers around mental health, including initial training on depressive symptom screening and cognitive behavioral counseling to address multiple dimensions of distress. Recent studies on provider perspectives on delivering mental health services in primary care settings in resource-limited areas such as Mexico [28] and the Dominican Republic [29] have also identified similar results. As resources are often limited, leveraging the strengths of existing staff by formally training those who are providing emotional support in informal ways, while reducing their others clinical tasks, may be an alternative. Comprehensive trainings can include topics related to stress-management, emotional well-being, and provide roleplaying exercises to improve providers’ self-efficacy to provide this type of support.

Providers also acknowledged their limited exposure to patients outside of their clinic appointments as a major barrier to addressing patient emotional wellbeing. Therefore, providers often considered patients’ family members as the ideal group to support patients. An important consideration is how access to familial support is gendered and may not be available for every patient. Providers noted that women with T2D often felt less supported in the home than men. This observation is consistent with T2D studies that have found that women report higher levels of depression, diabetes distress, emotional burden, and diabetes regimen-related stress than men [13, 30, 31]. Furthermore, the onus of who provides social support in the home often falls on women regardless of T2D status compared to men highlighting an imbalance of who is more likely to address emotional health needs for people with T2D [32, 33]. Establishing an emotional support network from peers and family members is essential for care, which may require training families on how to provide emotional support for those with T2D in the household. Overall, developing and strengthening social support networks can help remediate personal distress and improving the well-being of patients. Reflecting this, in the summer of 2018, the diabetes program that the providers we interviewed worked with, in collaboration with their newly formed Patient Associations, expanded the social support system with the formation of peer support groups, known as Groups of 5. The goal was to train peer leaders on diabetes management and social support provision. Future research and evaluation are needed to assess the impact of these efforts as well as the pathways of influence.

Some providers in our study reflected a willingness to provide more emotional support but also identified two key barriers: time and capacity to add additional activities to their workflow as a barrier [34, 35]. Therefore, there is a need to identify ways to integrate emotional support into the existing system of care. However, not every provider is equipped to do so. Key provider characteristics identified were patience, understanding, and a willingness to be patient-facing, all of which were identified as central to the role of *cooperadores*. A systematic review on facilitators to successful management of T2D in Latin America and the Caribbean found that positive attitude of health professionals, consistent trainings of health providers, using patient centered recommendations improved disease management [31]. Whereas more paternalistic approaches to provider-patient communication was a barrier to successful T2D management [31]. This is in line with our study focused on patients’ social support experiences, in which some patients mentioned that paternalistic tones from providers, particularly physicians, contributed to their feelings of distress [13]. Relatedly, a recent study focused on mental health services in the Dominican Republic from the perspective of providers found that non-mental health trained providers can carry stigmatizing attitudes towards patients presenting with mental health needs, which may influence their desire to manage their patient’s other conditions [29]. Thus, while the integration of emotional support in diabetes care is important, this integration requires staff who can provide that care in a patient-centered and empathetic way. In addition to improving ways to integrate emotional support with the clinic, developing and strengthening mental health referral systems by linking the clinics with local partners is another way of increasing the capacity of these community-based programs and improve the health and well-being of adults living with T2D.

Limitations of the study are that our results may not be transferable to other clinical contexts outside of the DR or primary clinics not focused on diabetes care. Although we interviewed physicians, nurses, and community health workers, additional interviews with physicians and nurses could have provided more clinician-specific perspectives, particularly clinics that function without collaborations with community health workers. However, the context of providing mental and emotional health care for adults with T2D can inform interventions and programs in similar resource-contrained settings.

## Conclusions

Providers were aware of the emotional burden their patients face managing T2D in rural areas of the Dominican Republic but lacked time and capacity to provide emotional support. Family was identified as the primary source of emotional support, followed by providers who had the identified skills and capacity to work with patients on emotional and mental health needs. Training on stress-management and emotional wellbeing specific to diabetes was identified as a needed next step to leverage existing providers towards being sources of emotional support for their patients. More research is needed on the feasibility and acceptability of implementing an integrated health model in rural community clinics.

## Supporting information

S1 Text Inclusivity in global research. (DOCX)
